# Diet quality in childhood: the Generation R Study

**DOI:** 10.1007/s00394-018-1651-z

**Published:** 2018-03-07

**Authors:** Laura A. van der Velde, Anh N. Nguyen, Josje D. Schoufour, Anouk Geelen, Vincent W. V. Jaddoe, Oscar H. Franco, Trudy Voortman

**Affiliations:** 1 Department of Epidemiology, Erasmus MC, University Medical Center, Rotterdam, the Netherlands; 2 The Generation R Study Group, Erasmus MC, University Medical Center, Rotterdam, the Netherlands; 3 Division of Human Nutrition, Wageningen University and Research, Wageningen, the Netherlands; 4 Department of Pediatrics, Erasmus MC, University Medical Center, Rotterdam, the Netherlands

**Keywords:** Diet quality, Dietary patterns, Determinants, Tracking, Validation, Epidemiology

## Abstract

**Purpose:**

We aimed to evaluate diet quality of 8-year-old children in the Netherlands, to identify sociodemographic and lifestyle correlates of child diet quality, and to examine tracking of diet quality from early to mid-childhood.

**Methods:**

For 4733 children participating in a population-based cohort, we assessed dietary intake using a validated food-frequency questionnaire at a median age of 8.1 years (interquartile range 8.0–8.2) (2011–2014). Based on dietary guidelines, we developed and validated a food-based diet quality score for children consisting of ten components (score 0–10): sufficient intake of vegetables; fruit; whole grains; fish; legumes; nuts; dairy; oils and soft fats; and low intake of sugar-containing-beverages; and high-fat and processed meat.

**Results:**

We observed a mean (± SD) diet quality score of 4.5 (± 1.2) out of a maximum of 10. On average, intake of legumes, nuts, and oils or soft fats was below recommendations, whereas intake of sugar-containing beverages and high-fat or processed meat was higher than recommended. The main factors associated with higher diet quality were higher maternal educational level (*β* = 0.29, 95% CI 0.21, 0.37 versus low education), higher household income (*β* = 0.15, 95% CI 0.05, 0.25 versus low income), no maternal smoking (*β* = 0.13, 95% CI 0.02, 0.25 versus current smoking), and less screen time (*β* = 0.31, 95% CI 0.24, 0.38)—all independent of each other. For children with available dietary data at age 1 year (*n* = 2608), we observed only weak tracking of diet quality from early to mid-childhood (Pearson’s *r* = 0.19, *k* = 0.11 for extreme quartiles).

**Conclusion:**

Overall diet quality of 8-year-old children did not conform to dietary guidelines, especially for children having more screen time, children of lower educated or smoking mothers, or from lower-income households.

**Electronic supplementary material:**

The online version of this article (10.1007/s00394-018-1651-z) contains supplementary material, which is available to authorized users.

## Background

A healthy diet during childhood is important for healthy growth and development [1], and may contribute to the prevention of obesity and chronic diseases later in life [2, 3]. Furthermore, dietary habits in childhood have been shown to track over time and are an important predictor of diet quality in adulthood [4]. Therefore, it is important to examine children’s dietary intake, to identify potential gaps between their actual and recommended intake, and to study determinants of diet, in order to develop targeted interventions focusing on groups with a high risk of poor dietary habits early in life.

Given the complexity of the human diet and the strong interactions between intake of different foods and nutrients, measuring overall dietary patterns is recommended as a complementary approach to measuring the intake of only single foods or nutrients [5, 6]. One way to study overall diet is by predefined diet quality scores, which are usually based on dietary guidelines [5, 7]. Although the use of diet quality indices in children has increased over the past years, Marshall et al. suggested in their systematic review that more prospective cohort studies evaluating diet quality in children and its impact on health are needed [8]. A few studies from different countries including the UK, Brazil, and the US assessed diet quality among school-age children [9–11]. However, because dietary habits and guidelines may differ between countries and cultures, it is important to use a diet quality score that assesses recommendations specific for the study population [12]. Previously, we developed a food-based diet quality score specifically for preschool children [13]. However, to date, no diet quality score is available for school-age children in the Netherlands, and factors related to diet quality have not been studied in this age category. Furthermore, previous studies reported tracking of diet from mid-childhood or adolescence to adulthood [2, 14], but information on changes in diet quality from early childhood to mid-childhood is scarce. This information is needed in order to establish whether dietary interventions could be efficient early in life [2].

Therefore, we aimed to evaluate overall diet quality of 8-year-old children participating in a large population-based cohort in the Netherlands. For this aim we developed a new food-based diet quality score based on current Dutch dietary guidelines [15], and we assessed the construct validity of this new diet quality score. This score can be applied in future studies to evaluate diet quality, to investigate associations between diet quality and health, and to support future dietary advice and interventions. Furthermore, we aimed to identify which parental and child sociodemographic and lifestyle factors, such as educational level, physical activity, and screen time correlate with diet quality of children and we aimed to investigate associations between diet quality at the ages of 1 and 8 years. This information can help to identify the best target groups and time frame for interventions to improve diet quality in children.

## Methods

### Study design and study population

**Fig. 1:**
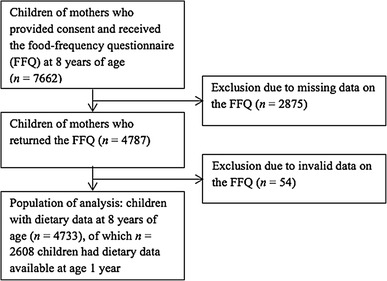
Flow chart of participants included in the study

This study was embedded in the Generation R Study, a multi-ethnic population-based prospective cohort from fetal life onward in Rotterdam, the Netherlands. Women living in the city of Rotterdam were enrolled during pregnancy. Children participating in this study were born between April 2002 and January 2006. The study was approved by the Medical Ethics Committee of Erasmus Medical Center and written informed consent was obtained from parents of all participating children [16]. A dietary questionnaire was sent to mothers who provided consent for follow-up when their child was around the age of 8 years (*n* = 7662). The questionnaire was returned for 4787 children (62.5%). After exclusion of subjects with invalid dietary data (*n* = 54), defined as a reported energy intake below 650 (*n* = 47) or above 3700 kcal/day (*n* = 7) (Additional file 1), valid dietary data were available for 4733 children (Fig. 1). Of all children with dietary data at the age of 8 years, 2608 children also had dietary data available at their age of 1 year.

### Dietary assessment

Dietary intake was assessed at a median age of 8.1 years (interquartile range (IQR) 8.0–8.2) using a validated semi-quantitative food-frequency questionnaire (FFQ) [17]. The FFQ was completed by the parents of the child, using the last 4 weeks as reference period. As explained in detail previously [17], the FFQ was developed based on results from a national food consumption survey in the Netherlands [18], which resulted in the selection of 71 food items relevant for the energy intake of 2- to 12-year-old children. Questions concerned the frequency of consumption and portion sizes of these foods, and for 27 food items additional questions were included about specific types or brands and preparation methods. Portion sizes were inquired for in natural units, household units, or grams; and parents were asked to measure the volume of glasses and cups regularly used by their child. Dietary intake data were cleaned and corrected based on detection of missing values, outliers of quantities, and inconsistencies using standardized algorithms developed for the FFQ [17]. Information on frequencies, types, and portion sizes was converted into grams of individual food items per day based on standard Dutch portion sizes, using SAS VoVris (Vovris V2.4, TNO, 1999–2006). Energy and nutrient intakes from foods were calculated using data from the Dutch Food Composition Table (NEVO 2001) with SAS Veves (Veves V2.2, TNO, 1999–2003).

The FFQ has been validated for energy intake among 4 to 6-year-old Dutch children (*n* = 30) using the doubly labeled water method [17]. The Pearson’s correlation between energy intake as estimated from the FFQ and energy expenditure measured with doubly labeled water was 0.62, indicating a reasonable capacity to rank subjects with respect to energy intake. Furthermore, no relevant intake-related bias was observed in the Bland–Altman plot. These findings indicate that the FFQ is a valid instrument for the assessment of energy intake in children [17].

### Construction of the diet quality score for school-age children

We constructed a food-based diet quality score based on dietary recommendations for children from the Netherlands Nutrition Center [15], thereby also taking into account the Dutch Guidelines for a Healthy Diet of 2015 [19], on which the Nutrition Center recommendations were based. We included ten components (i.e., food groups) in the diet quality score, of which eight were adequacy components (i.e., adequate intake is recommended) and two were moderation components (i.e., low intake is recommended). Only recommended food items were included for the adequacy components, and recommended food items were excluded for the moderation components (Table 1). The components, food items included, and their cut-off values were determined a priori, based on the recommendations of the Netherlands Nutrition Center for 8-year-old children [15], and were as follows: fruit (≥ 150 g/day), vegetables (≥ 150 g/day), whole grains (≥ 90 g/day), fish (≥ 60 g/week), legumes (≥ 84 g/week), nuts (≥ 15 g/day), dairy (≥ 300 g/day), oils and soft or liquid fats (≥ 30 g/day), sugar-containing beverages (≤ 150 g/day) and high-fat and processed meat (≤ 250 g/week) (Table 1).

**Table 1:** Components, cut-off values and included and excluded food items of the diet quality score

Component	Cut-off value^a^	Foods included in the diet quality score	Foods not included in the diet quality score
Fruit	≥ 150 g/d	Fresh fruit, frozen fruit, dried fruit (up to 20 g/d^b^), canned fruit without added sugar	Fruit juice, dried fruit (> 20 g/d), fruit products with added sugar
Vegetables	≥ 150 g/d	Fresh vegetables, frozen vegetables, canned vegetables^c^	–
Whole grains	≥ 90 g/d	Brown/whole-grain bread or crackers, whole-grain rice, whole-wheat pasta, whole-grain breakfast cereals without added sugar	White bread or crackers, white rice, white pasta, breakfast cereals with added sugar
Fish	≥ 60 g/w	Fish, canned fish, shellfish	Fish products containing < 70% fish (e.g., battered fish)
Legumes	≥ 84 g/w	Fresh, dried or canned legumes^c^	–
Nuts	≥ 15 g/d	Nuts, peanuts, peanut butter	Coated nuts
Dairy	≥ 300 g/d	Unsweetened, skimmed and semi-skimmed milk, yoghurt, or quark; dairy products without added sugar; buttermilk; low-fat cheese	Full-fat milk, yoghurt, or quark, dairy products with added sugar, full-fat cheese, whipped cream, ice cream
Oils and soft or liquid margarines	≥ 30 g/d	Vegetable oils, soft margarine (≤ 30% saturated fat of total fat), liquid cooking and frying fats	Hard margarine (> 30% saturated fat of total fat), hard cooking and frying fats, butter
Sugar-containing beverages	≤ 150 g/d^d^	Soft drinks, fruit juice, lemonade, fruit juice concentrates	Milk-based sugar-containing beverages^e^
High-fat and processed meat	≤ 250 g/w	Processed and/or high-fat meat (> 5% saturated fat)	Unprocessed low-fat meat (≤ 5% saturated fat)

*g/d* gram/day, *g/w* gram/week

^a^Based on the recommendations of the Netherlands Nutrition Center and Health Council of the Netherlands [15, 19]

^b^According to the guidelines, a maximum of 20 g/d of dried fruit was included in the fruit component

^c^Canned products with added salt or sugar were not excluded, as no distinction was made in the FFQ

^d^No quantitative recommendation was available for sugar-containing beverages, we chose a cut-off of 1 glass/d

^e^As milk-based beverages with added sugar can provide valuable nutrients and have a greater satiating effect compared to sweetened drinks containing sugar only, these were not included in the sugar-containing beverages component

Scoring of diet quality was performed by calculating the ratio of reported and recommended intake for each component [13]. These component scores were truncated at 1. For the adequacy components, this resulted in a minimum score of 0 points when these food items were not consumed and a maximum score of 1 point when the amount of the cut-off value or more was consumed. For example, a fruit intake of 60 g/day resulted in a score of 0.4 (60 divided by 150 g/day) for this component. For the moderation components, this scoring system was reversed, with higher scores reflecting lower intakes [e.g., a sugar-containing beverages intake of 45 g/day resulted in a score of 0.7 (1 − (45 divided by 150 g/day))]. Scores of the individual components were summed, resulting in a total score for diet quality ranging from 0 to 10 on a continuous scale, with a higher score indicating a better overall diet quality.

### Diet quality in infancy

To assess tracking of diet quality from infancy to mid-childhood, we used a previously defined diet quality score for infants. As described in detail elsewhere [13], information on dietary intake at the age of 1 year was collected with a semi-quantitative FFQ, which was developed specifically for this age group. This diet quality score consisted of ten components: vegetables; fruit; bread and cereals; rice, pasta, potatoes, and legumes; dairy; meat; fish; oils and fats; candy and snacks; and sugar-sweetened beverages. The scoring system for this diet quality score was similar to that of the diet quality score for 8-year-old children. The score ranged from 0 to 10 on a continuous scale with higher scores reflecting better adherence to dietary guidelines [13].

### Assessments of sociodemographic and lifestyle factors

Several sociodemographic and lifestyle factors were assessed for both the children and their parents. Information on date of birth and sex of the child was obtained from medical records and hospital registries. Ethnicity of the child was based on the country of birth of the parents, which was obtained with questionnaires at enrollment. If both parents were born in the Netherlands, the child was considered to have a Dutch ethnic background. If one parent was born outside of the Netherlands, the country of birth of that parent determined the child’s ethnicity. If both parents were born abroad, the country of birth of the mother determined the ethnicity of the child [16, 20]. Ethnicity was categorized according to the largest ethnic groups in our study population, which were Dutch, Moroccan, Turkish, Surinamese and Antillean, other Western, and other non-Western [13].

During follow-up visits of the participants to our research center at median ages of 6.0 years (IQR 5.9–6.2) and 9.7 years (IQR 9.6–9.9), we measured several child and maternal factors. Most measurements at these time points were strongly correlated. We used measurements taken at age 9.7 years for the main analyses, as this age was closest to the age of 8.1 years at dietary assessment. Child’s height and weight were measured to calculate their body mass index (BMI) (kg/m^2^), which was categorized into ‘underweight’, ‘normal weight’, or ‘overweight’ according to the Cole-criteria [21]. Questionnaires were used to assess time spent playing sports (i.e., any organized sports outside school hours), which was categorized into < 2, 2 to 4, or ≥ 4 h per week, and time spent watching television and/or using the computer (screen time), which was dichotomized into < 2 or ≥ 2 h per day [22]. At the same visits, we measured mothers’ height and weight to calculate BMI, which we categorized into ‘underweight’ (< 18.5), ‘normal weight’ (≥ 18.5–25), or ‘overweight’ (≥ 25) [21]. Information on other parental factors was assessed with questionnaires. Maternal smoking habits were categorized into: ‘never smoker’, ‘past smoker’, or ‘current smoker’. Maternal educational level was dichotomized into ‘no higher education’ or ‘higher education’, with higher education defined as completed higher vocational training or more. Net household income was dichotomized into < 2800 or ≥ 2800 euros per month [23].

### Statistical analysis

Child and parental characteristics were described as median (IQR) for continuous variables or percentage for categorical variables. Total diet quality score was described as mean with standard deviation (SD) and as percentage of children with the maximum score. Component scores and intake per component were described as median (IQR) and as percentage of children with the maximum score. Pearson’s correlations were used to assess correlations between the individual components of the diet quality score.

Linear regression models were used to identify sociodemographic and lifestyle correlates of diet quality. In these models, we examined children’s age, sex, ethnicity, BMI, physical activity, and screen time; maternal age, BMI, marital status, educational level, and smoking habits; and household income. The basic model was adjusted for total energy intake only; the multivariable model was additionally adjusted for all other sociodemographic and lifestyle variables that were examined in order to assess whether they were independent of each other. Associations of the diet quality score with intake of nutrients associated with a healthy diet were examined in order to assess the construct validity of the diet score (i.e., the degree to which the diet quality score measures a healthy diet) [24]. These associations were evaluated using Pearson’s correlations and partial Pearson’s correlations, controlling for energy intake.

Pearson’s correlations were also used to assess the association between diet quality at age 1 and 8 years. Tracking of diet quality score from age 1 year to age 8 years was assessed by determining to which extent children maintained their rank in the categories ‘lowest 25%’, ‘middle 50%’, or ‘highest 25%’. For this, a 3 × 3 matrix was constructed and a linear weighted Kappa statistic (*k*) was computed [25], with *k* < 0 indicating poor agreement and *k* 0.81–1.0 indicating almost perfect agreement [26].

Because the FFQ was developed and validated for a Dutch population, sensitivity analyses were performed among children with a Dutch ethnic background only (*n* = 3143). As non-response analysis, descriptive characteristics of children with valid dietary data (*n* = 4733) were compared to children with missing dietary data but who were eligible for dietary assessment (i.e., those who still participated in the study at the age of 8 years and who received the FFQ) (*n* = 2929). To reduce potential bias associated with missing data in our study, multiple imputation of missing data on sociodemographic and lifestyle factors was performed and ten independent datasets were created. Because similar effect estimates were found in analyses with imputed and unimputed data, pooled results after the multiple imputation were presented. All statistical analyses were performed using SPSS version 21.0 (IBM Corp., 2012, Armonk, NY). A two-sided *P* value of 0.05 was considered statistically significant.

## Results

### Subject characteristics

Characteristics of the children and their parents are described in Table 2. The majority of children had a Dutch ethnic background (66.4%). At the 9-year visit, median BMI of the children was 16.9 (IQR 15.7–18.4), with 80.7% of children having a normal weight, 7.2% underweight, and 12.1% overweight. More than half (51.6%) of the children had a screen time of ≥ 2 h per day. Median BMI of the mothers was 24.5 (IQR 22.3–27.5), with 55.1% of mothers having a normal weight and 44.1% overweight. The majority of mothers was highly educated (62.8%) and had never smoked (52.9%).

**Table 2:** Characteristics of study participants and their parents (*n* = 4733)

	Median (IQR), or percentage
**Child characteristics**	
Boy (%)	49.9
Ethnicity (%)	
Dutch	66.4
Other Western	9.4
Moroccan	3.6
Turkish	5.1
Surinamese and Antillean	7.2
Other non-Western	8.4
Age at FFQ (y)	8.1 (8.0–8.2)
BMI (kg/m^2^)	16.9 (15.7–18.4)
Underweight (%)	7.2
Normal weight (%)	80.7
Overweight (%)	12.1
Playing sports (%)	
< 2 h/w	31.9
2–4 h/w	40.7
≥ 4 h/w	27.4
Screen time (%)	
≥ 2 h/d	51.6
**Parental characteristics**	
Age mother at 9-year visit (y)	42.0 (39.0–44.6)
BMI mother (kg/m^2^)	24.5 (22.3–27.5)
Underweight (%)	0.8
Normal weight (%)	55.1
Overweight (%)	44.1
Marital status mother (%)	
Married/partner	88.1
Educational level mother (%)	
Higher education	62.8
Smoking mother (%)	
Never smoker	52.9
Past smoker	33.7
Current smoker	13.4
Household income per month (%)	
≥ 2800€	67.6

Values are medians (IQR) for continuous variables, and percentages for categorical variables, on the basis of imputed data (*n* = 10 imputations)

*IQR* interquartile range, *FFQ* food-frequency questionnaire, *y* year; *BMI* body mass index, *h/w* hours/week, *h/d* hours/day

Characteristics were similar before and after multiple imputation (Additional file 2). Of the 7662 children whose parents received the FFQ, children with missing dietary data (*n* = 2929) more often had a non-Dutch ethnic background and their mothers were on average younger, lower educated, had a higher BMI, and a lower household income than children with available dietary data (Additional file 3).

### Diet quality

Our diet quality score approximated a normal distribution with a mean (± SD) of 4.5 (± 1.2). None of the children reached the maximum possible diet quality score of 10. Median scores on most diet quality score components were around or below 0.5 out of a possible maximum of 1 (Table 3). For the adequacy components, median intakes of vegetables, legumes, nuts, dairy, and oils or soft or liquid fats were well below the cut-off values in our study population. For example, median daily vegetable intake was 79 g (IQR 49–123), whereas 150 g is recommended, resulting in a median component score of 0.53 (IQR 0.32–0.82) out of 1 for vegetables. Intakes of the two moderation components (sugar-containing beverages and high-fat and processed meat) exceeded the recommended intake in most children, with corresponding low scores. Median sugar-containing beverages intake, for example, was 323 g/day (IQR 180–524), with a median score of 0.0 (IQR 0.0–0.0) and only 12.8% of the children having a score above zero. Components with the highest median scores were whole grains (1.0 (IQR 0.72–1.0)), fruit (0.74 (IQR 0.51–1.0)), and fish (0.63 (IQR 0.0–1.0)). Correlations between the diet score components ranged from − 0.13 to 0.08. The scores were comparable between boys and girls (Additional file 4).

**Table 3:** Cut-offs values, actual intakes, and scores of the different diet quality score components

Component	Cut-off values	Unit	Intake	Score	% with a maximum score
Fruit	≥ 150	g/d	111 (77–167)	0.74 (0.51–1.00)	29.4
Vegetables	≥ 150	g/d	79 (48–123)	0.53 (0.32–0.82)	16.3
Whole grains	≥ 90	g/d	98 (65–131)	1.0 (0.72–1.00)	57.4
Fish^a^	≥ 60	g/w	38 (0–83)	0.63 (0.00–1.00)	36.0
Legumes^a^	≥ 84	g/w	18 (0–70)	0.21 (0.00-0.83)	21.1
Nuts^a^	≥ 15	g/d	3 (0–10)	0.20 (0.00-0.64)	10.6
Dairy	≥ 300	g/d	164 (54–298)	0.55 (0.18–0.99)	24.9
Oils and soft or liquid fats	≥ 30	g/d	11 (2–17)	0.37 (0.071–0.57)	2.7
Sugar-containing beverages^a^	≤ 150	g/d	323 (180–524)	0.00 (0.00–0.00)	2.1
High-fat and processed meat^a^	≤ 250	g/w	323 (218–453)	0.00 (0.00–0.13)	0.2

Values are median (IQR) Maximum score per component 1

*g/d* gram/day, *g/w* gram/week, *IQR* interquartile range

^a^A score of 0 was obtained by 27% of the participants for the fish component, 46.5% for the legumes component, 27.3% for the nuts component, 87.8% for the sugar-containing beverages component, and 68.2% for the meat component

Associations between the diet quality score and several nutrients were assessed to examine construct validity. We observed a positive correlation between the diet quality score and intakes of protein, mainly plant protein (*r* = 0.41), dietary fiber (*r* = 0.58), and *n* − 3 fatty acids (*r* = 0.24), and the score was  inversely correlated with intakes of saturated fat (*r * = − 0.11) and monosaccharides and disaccharides (*r* = − 0.11) (energy-adjusted, all *p* < 0.01). The score was also positively correlated with intake of all of the examined micronutrients (energy-adjusted, *r* = 0.16–0.55, all *p* < 0.01) (Additional file 5).

### Sociodemographic and lifestyle factors and the diet quality score

Associations between sociodemographic and lifestyle factors and the diet quality score are shown in Table 4. In the multivariable model, children with underweight had a lower diet quality score than children with a normal weight. Children with a screen time of ≥ 2 h per week had a 0.31 points lower diet quality score (95% CI − 0.38; − 0.24) than children with a screen time < 2 h per week, and children who played sports for 2–4 h per week had a 0.10 points higher diet quality (95% CI 0.02; 0.19) than those who played sports < 2 h per week, although no significant difference was found between playing sports for < 2 h per week versus ≥ 4 h per week. In the multivariable model, Moroccan children had a 0.29 points higher diet quality score (95% CI 0.10; 0.48) than children with a Dutch ethnicity, whereas in the basic model, without adjustment for the other factors, Turkish as well as Surinamese and Antillean children had a lower diet quality score than children with a Dutch ethnicity. Children’s sex or age at dietary assessment was not associated with the diet quality score in the basic and multivariable model.

**Table 4:** Associations between sociodemographic and lifestyle factors and the diet quality score

	Basic model^a^		Multivariable model^b^	
	*β* (95% CI)	*p* value	*β* (95% CI)	*p* value
**Child characteristics**				
Sex				
Boy	Reference			
Girl	− 0.01 (− 0.04; 0.03)	0.78	− 0.03 (− 0.10; 0.04)	0.41
Ethnicity				
Dutch	Reference			
Other Western	0.01 (− 0.10; 0.13)	0.83	0.03 (− 0.08; 0.14)	0.61
Moroccan	0.001 (− 0.18; 0.18)	1.00	0.29 (0.10; 0.48)	0.002
Turkish	− 0.43 (− 0.59; − 0.28)	< 0.001	− 0.11 (− 0.28; 0.06)	0.21
Surinamese and Antillean	− 0.21 (− 0.34; − 0.08)	0.002	0.05 (− 0.08; 0.19)0.47	
Other non-Western	− 0.11 (− 0.23; 0.01)	0.08	0.08 (− 0.04; 0.21)	0.19
Age at FFQ (y)	− 0.02 (− 0.16; 0.12)	0.77	0.06 (− 0.08; 0.20)	0.37
Energy intake (100 kcal/d)	0.10 (0.09; 0.10)	< 0.001	0.10 (0.09; 0.11)	< 0.001
Weight status				
Normal weight	Reference			
Underweight	− 0.13 (− 0.26; 0.003)	0.06	− 0.16 (− 0.29; − 0.03)	0.014
Overweight	− 0.17 (− 0.27; − 0.06)	0.003	0.01 (− 0.11; 0.12)	0.92
Playing sports				
< 2 h/w	Reference			
2–4 h/w	0.17 (0.07; 0.26)	< 0.001	0.10 (0.02; 0.19)	0.017
≥ 4 h/w	0.09 (− 0.02; 0.20)	0.11	0.04 (− 0.06; 0.15)	0.43
Screen time				
< 2 h/d	Reference			
≥ 2 h/d	− 0.39 (− 0.46; − 0.32)	< 0.001	− 0.31 (− 0.38; − 0.24)	< 0.001
**Parental characteristics**				
Age of mother at 9-year visit (y)	0.01 (0.01; 0.02)	< 0.001	0.001 (− 0.01; 0.01)	0.71
Weight status mother				
Normal weight	Reference			
Underweight	0.35(− 0.04; 0.75)	0.08	0.30 (− 0.08; 0.68)	0.12
Overweight	− 0.22 (− 0.29; − 0.14)	< 0.001	− 0.10 (− 0.17; − 0.02)	0.01
Marital status mother				
Married/partner	Reference			
No partner	− 0.23 (− 0.34; − 0.12)	< 0.001	− 0.03 (− 0.15; 0.09)	0.61
Educational level mother				
No higher education	Reference			
Higher education	0.44 (0.37; 0.51)	< 0.001	0.29 (0.21; 0.37)	< 0.001
Smoking status mother				
Never smoker	Reference			
Past smoker	0.05 (− 0.03; 0.14)	0.23	0.05 (− 0.03; 0.13)	0.26
Current smoker	− 0.26 (− 0.38; − 0.14)	< 0.001	− 0.13 (− 0.25; − 0.02)	0.027
Household income				
< 2800€/month	Reference			
≥ 2800€/month	0.32 (0.23; 0.40)	< 0.001	0.15 (0.05; 0.25)	0.004

*IQR* interquartile range, *FFQ* food-frequency questionnaire, *y* year, *BMI* body mass index, *kcal/d* kilocalorie/day, *h/w* hours/week, *h/d* hours/day

^a^Values are regression coefficients with 95% confidence intervals from linear regression analyses adjusted for total energy intake

^b^Values are regression coefficients with 95% confidence intervals from multivariable linear regression analyses including all variables presented in the table

Parental socioeconomic status was also associated with children’s diet quality: children of higher educated mothers or from households with a higher income had a higher diet quality score (Table 4). Independent of these socioeconomic factors, children of overweight mothers and children of mothers who were current smokers had a lower diet quality score than children of normal-weight or never-smoking mothers, respectively. Sensitivity analyses among Dutch children only showed similar effect estimates (Additional file 6).

### Tracking of diet quality from early to mid-childhood

For children with dietary data at both the ages of 1 year and 8 years (*n* = 2608), we observed a Pearson’s correlation of *r* = 0.19 for the diet quality score between both ages (*p* < 0.01). Significant correlations were also found between the seven individual diet quality score components that were examined at both time points (*r* = 0.11–0.23, all *p* < 0.01) (Additional file 7). A linear weighted kappa showed slight agreement between the diet quality scores at both ages [*k* = 0.11 (95% CI 0.08; 0.14)] for their rank in the lowest 25%, middle 50%, or highest 25% of the scores (Additional file 8).

## Discussion

We developed and validated a food-based diet quality score based on Dutch dietary guidelines to estimate overall diet quality of children. Using this score, we evaluated diet quality of over 4700 children at the age of 8 years in a population-based cohort in the Netherlands. We observed that diet quality in this population was suboptimal and none fully adhered to the guidelines. Factors that correlated with a higher diet quality in this group were, amongst others, a higher socioeconomic status and no maternal smoking. We observed only weak tracking of diet quality between the ages of 1 and 8 years.

### Interpretation of findings and comparison with previous research

Diet quality was suboptimal in our study population of 8-year-old children. This is consistent with studies in the US, Brazil, and the UK that showed less than optimal diet quality in similarly aged children [9–11]. Compared to the American population aged 7–9 years [9], level of adherence was similar for the fruit and vegetable components, however, for the dairy component adherence was lower in our study population. This might be explained by the difference in scoring, as we only included recommended food items in the dairy component, whereas in the US-based study all dairy items were included [9].

As expected from previous studies [27], socioeconomic status was positively associated with diet quality. A strong association was observed particularly for maternal educational level, independent of household income and other factors. Previous studies indicated that individuals with a higher educational level could have more nutritional knowledge [4, 27, 28]; our study suggests that this also translates to the diet provided to their children. Furthermore, families with a higher income may be more able to buy healthy, more expensive, food products [29, 30], explaining our association of household income with child diet quality, independent of educational level. A previous study among households in Canada showed that access to dairy, fruit, and vegetables, which are food groups that positively contribute to our diet score, may be constrained by low income irrespective of educational level [31]. Unfortunately we did not assess food security in our study, which may partly explain the association between socioeconomic status and diet quality found in our study.

The negative association of maternal smoking with diet quality score is consistent with previous research among 515 children aged 2–17 years in the U.S., which showed that children from low-income families with parents who smoked, had a poorer diet quality than children from low-income families with non-smoking parents [32]. We also observed a negative association between maternal overweight and child’s diet quality score, which is in line with a previous study among 1640 children aged 3 years in the UK [33]. These findings for maternal smoking and overweight suggest that an unhealthy lifestyle of the mother negatively influences their child’s diet quality, independent of socioeconomic status.

Independent of these maternal factors, we also found an association between children’s lifestyle and their diet quality. Being more physically active and having less screen time were associated with a higher diet quality in children, which is consistent with previous research, that showed that sedentary behavior is associated with a less healthy diet [27, 33]. However, we did not observe this association for the group with the highest levels of physical activity. Finally, in our fully adjusted models, we observed that children with a Moroccan ethnic background had a higher diet quality score than those with a Dutch ethnicity. Children with a Moroccan ethnicity had higher intakes of fish, legumes, and nuts and a lower high-fat and processed meat intake, suggesting a more Mediterranean-style diet [34].

In our study population, we found only weak tracking of the diet quality score and its individual components between the ages of 1 and 8 years. Studies on tracking of diet from early life to later childhood are limited [35]. One previous study found moderate to fair tracking of the intakes of fruit, vegetables, and sugar-sweetened beverages from the age of 18 months to 7 years [35]. A review by Nicklaus and Remy (2013), showed moderate tracking of eating habits after the first year of life [36]. Combined, these results suggest that tracking of diet may start after the age of 1 year.

### Methodological considerations of the diet quality score

The diet quality score was positively associated with intake of micronutrients, indicating adequate construct validity, since dietary recommendations are, amongst others, developed to provide a sufficient supply of nutrients [24]. We included both healthy and unhealthy components in the score, which may better capture overall diet quality than including healthy or unhealthy components only, as eating healthy foods is not necessarily inversely related to eating unhealthy foods [37]. Further research is needed to examine whether this combined score is indeed associated with child health. Another strength of our diet score is the use of cut-off values based on current dietary recommendations instead of using a population-specific cut-off value such as a population-specific median intake, which may not be related to an actual healthy intake level [24]. Finally, a strength of our diet quality scoring system is that we used a continuous scale, which provides more detail and is more accurate in ranking children with respect to diet quality than a dichotomous scoring system [24].

Constructing an overall diet quality index involves many choices [24]. Although it may have been preferred to ascribe greater weights to components that have a greater effect on health, not enough information on the overall health effects of individual components was available, so we chose not to apply any weighting. In addition to the number and weights of components, another aspect to consider is the type of components included in the diet index. Most diet indices are based on intake of nutrients, food groups, or a combination of these, and some indices also include measures of dietary variety [24, 38]. We chose to construct our diet quality score on the basis of intake of food groups only, in line with the Dutch dietary guidelines, but we also observed positive associations of the diet score with intake of micronutrients, suggesting it represents an overall healthy diet. When diet quality score components are similar to each other or when they are strongly correlated, they contribute more heavily to the score [24]. However, in our diet quality score, we observed low correlations between the diet score components (*r* − 0.13 to 0.08). Finally, because our diet quality score is based on Dutch recommendations, important food groups may be absent for children with another ethnic background. However, the Dutch recommendations are comparable to recommendations in other countries [39]. Furthermore, a systematic review conducted by Gilbert and Khokhar (2008) showed that after moving to a Western country, the majority of ethnic groups change their eating habits to a more Western diet [40]. Also, we did not find major differences in diet score between the different ethnic groups in our population, suggesting that the Dutch recommendations and our diet score were also suitable for the study participants with another ethnicity.

### Strengths and limitations

Major strengths of the Generation R Study, in which we applied our diet quality score, are the population-based prospective cohort design and the large number of subjects. Also, we had information available on many parental and child sociodemographic and lifestyle factors for which we could examine their correlation with diet quality. However, there may be other correlates of diet quality that were not assessed in our study. Unfortunately, we had no detailed information on physical activity of the children. We used the amount of time participating in organized sports as a proxy for physical activity, which may not be an optimal measure, because it does not take into account other sources of physical activity. In addition, not all correlates were assessed at the same moment as dietary assessment, which may have influenced the associations. However, there was a high correlation of most variables throughout childhood, and we expect that any changes in these correlates are only limited. Therefore, we chose the time point that was the closest to our moment of dietary assessment. Furthermore, non-response analyses showed that non-responders to the FFQ more often had characteristics associated with a lower diet quality score, such as a lower educational level, suggesting that diet quality might be even lower in the children in Rotterdam than observed in the study population for which we had data available.

Another limitation of the study was the assessment of dietary intake with an FFQ. Limitations of FFQs in general are that they contain a limited amount of food items, and recollection of the consumed foods and portion sizes can be sources of error [41]. The FFQ used in our study was validated against the doubly labeled water method, regarded as the golden standard for the determination of total energy expenditure in free-living subjects, and this validation showed a reasonable capacity of the FFQ to rank subjects with respect to energy intake [17]. However, the FFQ was not validated for the intake of specific foods or food groups. Finally, for our analyses on tracking of diet quality, a limitation was that diet quality was not scored in exactly the same manner at the ages of 1 and 8 years and that no data on dietary intake were available for the period in between these two age categories.

### Implications

The results of our study suggest that overall diet quality among 8-year-old children in our study population in an urban multi-ethnic setting in the Netherlands is suboptimal and that none of the children fully adhered to the dietary guidelines. This is undesirable as a healthy diet is important for healthy growth and development of the child [1]. However, future research is needed to assess whether a higher overall adherence to the dietary guidelines is indeed associated with a better health and to evaluate diet quality in other populations of children. Although the observed effect estimates for the correlates of diet quality were relatively small on an individual level, these may be relevant for public health strategies. We observed for example that children from higher educated mothers had an 0.3 higher diet quality score (scale 0–10) than children whose mothers had not completed higher education. Most of the observed correlates of diet quality in our study are in line with previous research. Consistent with other studies, we found low socioeconomic status to be a strong predictor of a lower diet quality [27], emphasizing the need to target child dietary interventions especially to families with a lower socioeconomic status. Interventions should focus on promotion of healthy food products and increase the accessibility of these foods for these groups. Additionally, interventions should also discourage the consumption of unhealthy food products. Adherence to the recommendations was particularly low for the moderation components in our study population, underlining the importance of discouraging the intake of sugar-containing beverages and high-fat and processed meat. As previous evidence showed tracking of diet between mid-childhood and adulthood [14], dietary interventions targeted at children are expected to not only improve diet quality during childhood, but also their diet quality into adulthood. However, we observed only weak tracking of diet quality from early to mid-childhood. Therefore, further research is needed to establish the optimal age and also the best target groups (e.g., children, parents, and/or schools) for dietary interventions in order to improve long-term diet quality.

## Conclusion

To conclude, in this large population-based cohort in the Netherlands, we observed that diet quality of 8-year-old children was suboptimal, which indicates that they do not meet the current dietary guidelines. Particularly the intake of legumes, nuts, and oils or soft or liquid fats was too low, whereas the intake of sugar-containing beverages and high-fat and processed meat was too high. Main sociodemographic and lifestyle factors that correlated with a higher diet quality were a higher maternal education, a higher household income, no maternal smoking, and less time spent on watching television or using a computer of the child. Tracking of diet quality from the age of 1 to 8 years was weak.

## Electronic supplementary material

Below is the link to the electronic supplementary material.

Supplementary material 1 (DOCX 79 KB)
