# Supplementary material for: Diet quality in childhood: the Generation R Study
Source: Eur J Nutr. 2018 Mar 7;58(3):1259–69. doi: 10.1007/s00394-018-1651-z (PMC6499873; doi:10.1007/s00394-018-1651-z)
Supplement: Supplementary file 1 — Supplementary material 1 (DOCX 79 KB) [file 394_2018_1651_MOESM1_ESM.docx]

# Additional material

**Additional file 1** Data cleaning of dietary intake data

Dietary intake data obtained with the FFQ were cleaned and corrected based on detection of missings, outliers of quantities, and inconsistencies through standardized algorithms developed specifically for this FFQ (Dutman et al, 2011). The following standard procedures were applied: Where a frequency indicated that an item was consumed, but a quantity or type was missing, the missing value was replaced with a predefined default for that particular item. Where a quantity was filled in, but a frequency was missing, the frequency was assumed to be zero. Remaining cases with a reported energy intake below 650 or above 3700 kcal/day were excluded (1.1%). These cut-offs were based on stemplots of the lower and higher end of the energy intake distributions of the study population and were placed where the monotonous increase and decrease of the values was disrupted (Dutman et al, 2011, Figures S1 and S2).

**Figure S1** Stemplot of the lowest end of the energy intake distribution
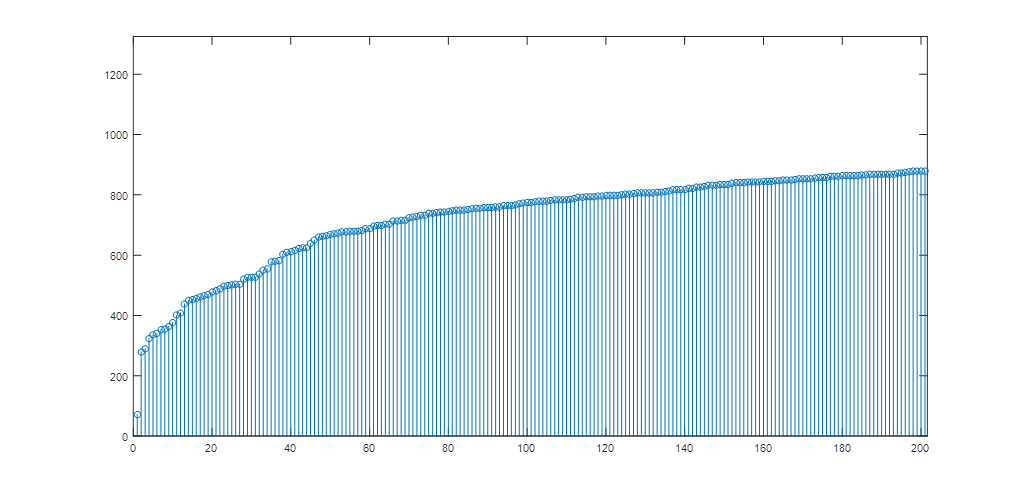


Energy intake (kcal/d)

**Figure S2** Stemplot of the highest end of the energy intake distribution
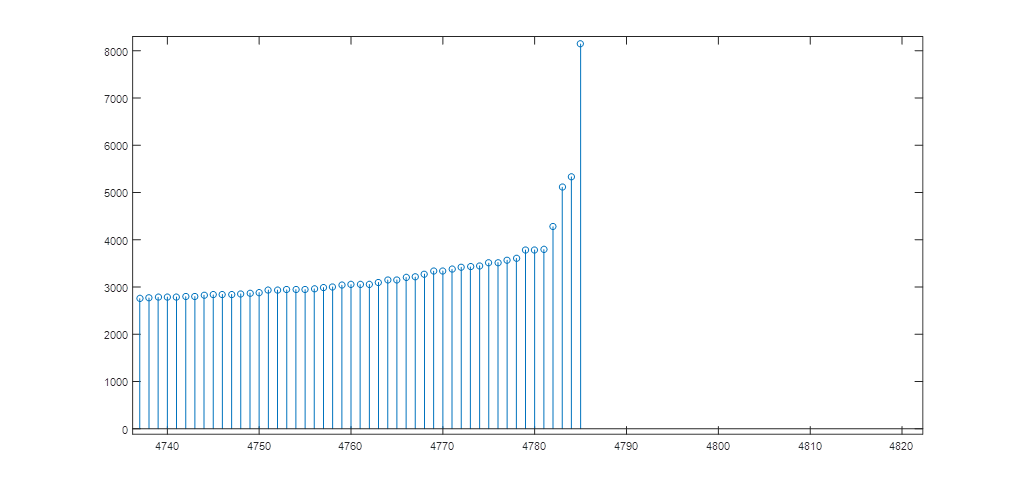


Energy intake (kcal/d)

**Additional file 2** Characteristics of study participants and their parents in imputed and unimputed data

|  | | **Complete data, n data** | | **Missing,**  **n (%)** | **Unimputed data** | | **Imputed data** |
| --- | --- | --- | --- | --- | --- | --- | --- |
| **Child characteristics** | | | | | | |  |
| Boy (%) | | 4,732 | 1 (0.0%) | | | 49.9 | 49.9 |
| Ethnicity (%) | | 4,707 | 26 (0.5% | | |  |  |
|  | Dutch |  |  | | | 66.8 | 66.4 |
|  | Other Western |  |  | | | 9.3 | 9.4 |
|  | Moroccan |  |  | | | 3.5 | 3.6 |
|  | Turkish |  |  | | | 5.0 | 5.1 |
|  | Surinamese and Antillean |  |  | | | 7.1 | 7.2 |
|  | Other non-Western |  |  | | | 8.3 | 8.4 |
| Age at FFQ (y) | | 4,724 | 9 (0.2%) | | | 8.1 (8.0-8.2) | 8.1 (8.0-8.2) |
| BMI (kg/m^2^) | | 3,991 | 742 (15.7%) | | | 16.8 (15.6-18.3) | 16.9 (15.7-18.4) |
|  | Underweight (%) |  |  | | | 8.1 | 7.2 |
|  | Normal weight (%) |  |  | | | 78.5 | 80.7 |
|  | Overweight (%) |  |  | | | 13.4 | 12.1 |
| Playing sports (h/w) | | 3,431 | 1,302 (27.5%) | | |  |  |
|  | <2 |  |  | | | 31.4 | 31.9 |
|  | 2-4 |  |  | | | 45.7 | 40.7 |
|  | ≥4 |  |  | | | 22.9 | 27.4 |
| Screen time (h/d) | | 3,415 | 1,318 (27.8%) | | |  |  |
|  | ≥2 |  |  | | | 50.1 | 51.6 |
| **Parental characteristics** | | | | | | |  |
| Age mother at 9-year visit (y) | | 3,958 | | 775 (16.4%) | | 42.1 (39.1-44.7) | 42.0 (39.0-44.6) |
| BMI mother (kg/m2) | | 3,958 | | 775 (16.4%) | | 24.4 (22.2-27.6) | 24.5 (22.3-27.5) |
|  | Underweight (%) |  | |  | | 0.9 | 0.8 |
|  | Normal weight (%) |  | |  | | 55.6 | 55.1 |
|  | Overweight (%) |  | |  | | 43.5 | 44.1 |
| Marital status mother (%) | | 4,441 | | 292 (6.2%) | |  |  |
|  | Married/ partner |  | |  | | 88.9 | 88.1 |
| Educational level mother (%) | | 4,450 | | 283 (6.0%) | |  |  |
|  | Higher education |  | |  | | 64.1 | 62.8 |
| Smoking mother (%) | | 4,107 | | 626 (13.2%) | |  |  |
|  | Never smoker |  | |  | | 53.5 | 52.9 |
|  | Past smoker |  | |  | | 33.7 | 33.7 |
|  | Current smoker |  | |  | | 12.9 | 13.4 |
| Household income per month (%) | | 3,633 | | 1,100 (23.2%) | |  |  |
|  | ≥2800 € |  | |  | | 71.0 | 67.6 |

*Values are median (IQR) or valid percentage.*

*IQR= interquartile range; FFQ=Food-frequency questionnaire; y= year; BMI=Body mass index; kg/m2= kilogram/ meter^2^; h/w= hours/ week; h/d= hours/ day.*

**Additional file 3** Child and parental characteristics of responders (n=4,733) and non-responders (n=2,929) to the food-frequency questionnaire (FFQ)

|  | | **n** | **Responders** | **n** | **Non-responders** |
| --- | --- | --- | --- | --- | --- |
| **Child characteristics** | |  |  |  |  |
| Boy (%) | | 4,732 | 49.9 | 2,929 | 50.8 |
| Ethnicity (%) | | 4,707 |  | 2,713 |  |
|  | Dutch |  | 66.8 |  | 42.3 |
|  | Other Western |  | 9.3 |  | 7.0 |
|  | Moroccan |  | 3.5 |  | 10.0 |
|  | Turkish |  | 5.0 |  | 11.0 |
|  | Surinamese and Antillean |  | 7.1 |  | 16.9 |
|  | Other non-Western |  | 8.3 |  | 12.8 |
| BMI (kg/m^2^) | | 3,991 | 16.8 (15.6-18.3) | 1,503 | 17.5 (16.0-19.9) |
|  | Underweight (%) |  | 8.1 |  | 6.5 |
|  | Normal weight (%) |  | 78.5 |  | 68.5 |
|  | Overweight (%) |  | 13.4 |  | 25.0 |
| Playing sports (h/w) | | 3,431 |  | 813 |  |
|  | <2 |  | 31.4 |  | 38.9 |
|  | 2-4 |  | 45.7 |  | 37.4 |
|  | ≥4 |  | 22.9 |  | 23.7 |
| Screen time (h/d) | | 3,415 |  | 811 |  |
|  | ≥2 |  | 50.1 |  | 62.9 |
| **Parental characteristics** | |  |  |  |  |
| Age mother at 9-year visit (y) | | 3,958 | 42.1 (39.1-44.7) | 1,513 | 39.9 (35.7-43.5) |
| BMI mother (kg/m^2^) | | 3,958 | 24.4 (22.2-27.6) | 1,513 | 25.8 (23.0-30.4) |
|  | Underweight (%) |  | 0.9 |  | 0.6 |
|  | Normal weight (%) |  | 55.6 |  | 42.2 |
|  | Overweight (%) |  | 43.5 |  | 57.2 |
| Marital status mother (%) | | 4,441 |  | 1,730 |  |
|  | Married/ partner |  | 88.9 |  | 78.7 |
| Educational level mother (%) | | 4,450 |  | 1,709 |  |
|  | Higher education |  | 64.1 |  | 38.5 |
| Smoking mother (%) | | 4,107 |  | 1,084 |  |
|  | Never smoker |  | 53.5 |  | 52.3 |
|  | Past smoker |  | 33.7 |  | 26.9 |
|  | Current smoker |  | 12.9 |  | 20.8 |
| Household income per month (%) | | 3,633 |  | 916 |  |
|  | ≥2800 € |  | 71.0 |  | 45.0 |

*Values are median (IQR) or valid percentages on the basis of unimputed data.*

*IQR= Interquartile range; FFQ=Food-frequency questionnaire; y= year; BMI=Body mass index; kg/m2= kilogram/ meter^2^; h/w= hours/ week; h/d= hours/ day.*

**Additional file 4** Median intake per diet quality score component and median score per diet quality score component (maximum score is 1) for boys (n=2364) and girls (n=2369) separately

|  |  | | Intake | | Score | |
| --- | --- | --- | --- | --- | --- | --- |
| Component | Unit | Cut-off values | Boys | Girls | Boys | Girls |
| Fruit | g/d | ≥ 150 | 111 (77-165) | 111 (77-167) | 0.74 (0.51-1.00) | 0.74 (0.51-1.00) |
| Vegetables | g/d | ≥ 150 | 77 (47-122) | 80 (51-123) | 0.51 (0.31-0.82) | 0.53 (0.34-0.82) |
| Whole-grains | g/d | ≥ 90 | 101 (65-135) | 98 (65-130) | 1.00 (0.72-1.00) | 1.00 (0.72-1.00) |
| Fish | g/w | ≥ 60 | 38 (0-95) | 38 (0-79) | 0.63 (0.00-1.00) | 0.63 (0.00-1.00) |
| Legumes | g/w | ≥ 84 | 35 (0-70) | 18 (0-70) | 0.42 (0.00-0.83) | 0.21 (0.00-0.83) |
| Nuts | g/d | ≥ 15 | 4 (0-11) | 2 (0-8) | 0.29 (0.00-0.70) | 0.14 (0.00-0.53) |
| Dairy | g/d | ≥ 300 | 182 (56-319) | 152 (54-284) | 0.61 (0.19-1.00) | 0.51 (0.18-0.95) |
| Oils and soft or liquid fats | g/d | ≥ 30 | 11 (2-22) | 11 (2-17) | 0.37 (0.07-0.74) | 0.37 (0.07-0.56) |
| Sugar-containing beverages | g/d | ≤ 150 | 335 (193-557) | 311 (169-506) | 0.00 (0.00-0.00) | 0.00 (0.00-0.00) |
| High-fat and processed meat | g/w | ≤ 250 | 340 (232-482) | 306 (203-430) | 0.00 (0.00-0.07) | 0.00 (0.00-0.19) |

*Values are median (IQR).*

*Energy intake (kcal/d) was 1531 (IQR 1299-1763) among boys and 1391 (IQR 1184-1606) among girls.*

*g/d= gram/ day; g/w= gram/ week; IQR= Interquartile range; kcal/d= kilocalorie/ day.*

**Additional file 5** Associations between the diet quality score and intake of nutrients

|  | | Pearson correlation (r)  Unadjusted | Pearson correlation (r)  Adjusted for energy intake |
| --- | --- | --- | --- |
| **Macronutrients:** | |  |  |
| Fat (g) | | 0.26 | -0.02 |
|  | Saturated fat (g) | 0.20 | -0.11 |
|  | N-3 fatty acids (mg) | 0.26 | 0.24 |
| Protein (g) | | 0.40 | 0.29 |
|  | Animal protein (g) | 0.24 | 0.05 |
|  | Plant protein (g) | 0.49 | 0.41 |
| Carbohydrates (g) | | 0.24 | -0.11 |
|  | Monosaccharides and disaccharides (g) | 0.15 | -0.11 |
|  | Dietary fiber (g) | 0.62 | 0.58 |
| **Micronutrients:** | |  |  |
| Vitamin B 1 (mg) | | 0.46 | 0.36 |
| Vitamin B 2 (mg) | | 0.38 | 0.25 |
| Vitamin B 3 (niacin) (mg) | | 0.42 | 0.31 |
| Vitamin B 6 (mg) | | 0.43 | 0.32 |
| Vitamin B 12 (µg) | | 0.30 | 0.18 |
| Vitamin C (mg) | | 0.29 | 0.16 |
| Vitamin D (µg) | | 0.40 | 0.30 |
| Calcium (mg) | | 0.39 | 0.27 |
| Copper (mg) | | 0.54 | 0.52 |
| Iron (mg) | | 0.45 | 0.36 |
| Magnesium (mg) | | 0.55 | 0.55 |
| Phosphorus (mg) | | 0.48 | 0.44 |
| Selenium (µg) | | 0.37 | 0.24 |
| Zinc (mg) | | 0.41 | 0.31 |

*All r-values were statistically significant at the 0.05 level, except for total fat (adjusted for energy intake): p=0.16.*

*g= gram; mg= milligram; µg= microgram.*

**Additional file 6** Associations between sociodemographic and lifestyle factors and the diet quality score among children with a Dutch ethnic background (n=3,143)

|  | | | **Basic model^a^** | | **Multivariable model^b^** | |
| --- | --- | --- | --- | --- | --- | --- |
|  | | | β (95 % CI) | p-value | β (95 % CI) | p-value |
| **Child characteristics** | | | | | | |
| Sex | | |  |  |  |  |
|  | | Boy | *Reference* |  |  |  |
|  | | Girl | 0.02 (-0.07; 0.10) | 0.71 | -0.02 (-0.10; 0.06) | 0.63 |
| Age at FFQ (y) | | | -0.09 (-0.27; 0.09) | 0.34 | 0.01 (-0.17; 0.19) | 0.91 |
| Energy intake (kcal) | | | 0.001 (0.001; 0.001) | <0.001 | 0.001 (0.001; 0.001) | <0.001 |
| BMI | | |  |  |  |  |
|  | | Normal weight | *Reference* |  |  |  |
|  | | Underweight | -0.12 (-0.28; 0.03) | 0.12 | -0.15 (-0.30; -0.001) | 0.052 |
|  | | Overweight | -0.15 (-0.30; -0.003) | 0.054 | 0.002 (-0.15; 0.15) | 0.98 |
| Playing sports (h/w) | | |  |  |  |  |
|  | | <2 | *Reference* |  |  |  |
|  | | 2-4 | 0.10 (-0.01; 0.20) | 0.06 | 0.05 (-0.05; 0.15) | 0.28 |
|  | | ≥4 | 0.06 (-0.07; 0.18) | 0.38 | 0.01 (-0.11; 0.13) | 0.87 |
| Screen time (h/d) | | |  |  |  |  |
|  | | <2 | *Reference* |  |  |  |
|  | | ≥2 | -0.39 (-0.48; -0.31) | <0.001 | -0.32 (-0.41; -0.23) | <0.001 |
| **Parental characteristics** | | | | | | |
| Age mother at 9-year visit (y) | | | 0.01 (0.001; 0.02) | 0.029 | 0.001 (-0.01; 0.01) | 0.82 |
| BMI mother | | |  |  |  |  |
|  | Normal weight | | *Reference* |  |  |  |
|  | Underweight | | 0.55 (0.10; 1.00) | 0.018 | 0.45 (0.011; 0.89) | 0.045 |
|  | Overweight | | -0.22 (-0.31; -0.13) | <0.001 | -0.13 (-0.22; -0.04) | 0.004 |
| Marital status mother | | |  |  |  |  |
|  | | Married/ partner | *Reference* |  |  |  |
|  | | No partner | -0.17 (-0.32; -0.02) | 0.029 | 0.01 (-0.15; 0.18) | 0.88 |
| Educational level mother | | |  |  |  |  |
|  | | No higher education | *Reference* |  |  |  |
|  | | Higher education | 0.47 (0.38; 0.55) | <0.001 | 0.33 (0.23; 0.42) | <0.001 |
| Smoking mother | | |  |  |  |  |
|  | | Never smoker | *Reference* |  |  |  |
|  | | Past smoker | 0.03 (-0.07; 0.13) | 0.52 | 0.06 (-0.03; 0.15) | 0.21 |
|  | | Current smoker | -0.31 (-0.46; -0.17) | <0.001 | -0.16 (-0.30; -0.02) | 0.024 |
| Household income per month | | |  |  |  |  |
|  | | <2800 € | *Reference* |  |  |  |
|  | | ≥2800 € | 0.27 (0.17; 0.38) | <0.001 | 0.12 (0.003; 0.24) | 0.045 |

*^a^Values are regression coefficients with 95% confidence intervals from linear regression analyses adjusted for total energy intake*

*^b^Values are regression coefficients with 95% confidence intervals from multivariable linear regression analyses including all variables presented in the table. CI= Confidence interval; FFQ=Food-frequency questionnaire; y= year; kcal= kilocalorie; BMI=Body mass index; h/w= hours/ week; h/d= hours/ day.*

**Additional file 7** Associations between the diet quality score and its components at the age of 1 y and at the age of 8 y (n=2,608)

| Component | Pearson correlation (r) |
| --- | --- |
| Fruit | 0.20 |
| Vegetables | 0.11 |
| Whole-grains | 0.19 |
| Fish | 0.23 |
| Dairy | 0.14 |
| Oils and unsaturated fats | 0.19 |
| Sugar-containing beverages | 0.12 |
| Total | 0.19 |

*All significant at 0.01 level*

*The components ‘legumes’ and ‘nuts’ were not incorporated in the diet quality score at age 1 year, and the ‘meat’ component was an adequacy component (low-fat unprocessed meat) in the preschool score and a moderation component (high-fat and processed meat) in the childhood score, therefore no tracking was assessed for these components.*

**Additional file 8** Tracking matrix constructed for the calculation of weighted Kappa statistic for diet quality score at age 1 y and age 8 y among children with dietary data at both time points (n=2,607)

|  | Lowest 25% at 8 y | Middle 50% at 8 y | Highest 25% at 8 y |
| --- | --- | --- | --- |
| Lowest 25% at 1 y | 201 | 339 | 112 |
| Middle 50% at 1 y | 302 | 653 | 348 |
| Highest 25% at 1 y | 104 | 317 | 231 |

*The entry in a specific cell indicates the number of subjects belonging to the corresponding classes at age 1 y and age 8 y. y= year.*
